# A knowledge-inherited learning for intelligent metasurface design and assembly

**DOI:** 10.1038/s41377-023-01131-4

**Published:** 2023-03-30

**Authors:** Yuetian Jia, Chao Qian, Zhixiang Fan, Tong Cai, Er-Ping Li, Hongsheng Chen

**Affiliations:** 1 ZJU-UIUC Institute, Interdisciplinary Center for Quantum Information, State Key Laboratory of Extreme Photonics and Instrumentation, Zhejiang University, Hangzhou, 310027 China; 2 ZJU-Hangzhou Global Science and Technology Innovation Center, Key Lab. of Advanced Micro/Nano Electronic Devices & Smart Systems of Zhejiang, Zhejiang University, Hangzhou, 310027 China; 3 Jinhua Institute of Zhejiang University, Zhejiang University, Jinhua, 321099 China; 4 Air and Missile Defense College, Air Force Engineering University, Xi’ an, 710051 China; 5 Shaoxing Institute of Zhejiang University, zhejiang University, Shaoxing, 321000, China

**Keywords:** Metamaterials, Sub-wavelength optics

## Abstract

Recent breakthroughs in deep learning have ushered in an essential tool for optics and photonics, recurring in various applications of material design, system optimization, and automation control. Deep learning-enabled on-demand metasurface design has been the subject of extensive expansion, as it can alleviate the time-consuming, low-efficiency, and experience-orientated shortcomings in conventional numerical simulations and physics-based methods. However, collecting samples and training neural networks are fundamentally confined to predefined individual metamaterials and tend to fail for large problem sizes. Inspired by object-oriented C++ programming, we propose a knowledge-inherited paradigm for multi-object and shape-unbound metasurface inverse design. Each inherited neural network carries knowledge from the “parent” metasurface and then is freely assembled to construct the “offspring” metasurface; such a process is as simple as building a container-type house. We benchmark the paradigm by the free design of aperiodic and periodic metasurfaces, with accuracies that reach 86.7%. Furthermore, we present an intelligent origami metasurface to facilitate compatible and lightweight satellite communication facilities. Our work opens up a new avenue for automatic metasurface design and leverages the assemblability to broaden the adaptability of intelligent metadevices.

## Introduction

The awakened wave of machine learning has swept across a variety of scientific areas, ranging from the mainstream applications of image recognition and language translation to the emerging disciplines of neuroscience and quantum mechanics^1^. In optics and photonics, we have been witnessing the interaction of machine learning transforming the way we design new photonic structures, unearth latent physical laws, and develop intelligent photonic devices^2–5^. Metamaterials and their planar equivalences, metasurfaces, may be the most thought-provoking. By rationally designing artificially subwavelength structures and spatiotemporal layouts, metamaterials and metasurfaces can provide an unprecedented ability to manipulate the electromagnetic (EM) wavefront at will, and thus, facilitate a panoply of exciting phenomena and novel devices^6–9^. Recently, a growing interest has been fueled on inverse structural/material design and forward EM response prediction^10–14^. Conventionally, these tasks are executed by a large number of EM numerical simulations. However, it necessitates iterative and lengthy calculations of Maxwell’s equations^15,16^, and even worse, the ultimate design outcomes are innately flawed in a trial-and-error manner. In this respect, machine learning is heralded as a promising method to unlock elusive light-metamaterial interactions with powerful nonlinear fitting and generalizability. We have seen extensive literature across diversified metamaterials^17,18^, plasmonic nanostructures^19,20^, and photonic crystals^21,22^, spawning data-driven approaches complementary or superior to conventional methods.

Two key problems must be deciphered in all machine learning-based metamaterial designs, i.e., data collection and algorithm modeling^23^. Data to machine learning is like the fuel to an engine. To enable a powerful driving force, much effort has been inaugurated to enlarge the training dataset. However, it was soon realized that such a method is prohibitively time-consuming and computationally expensive, especially with the increase in design dimension and metamaterial scale^24,25^. To mitigate this dilemma, data clustering, feature extraction, data denoising, and related techniques have been extensively leveraged to augment the data utilization efficiency. Meanwhile, scientists strive to introduce novel network structures, such as transfer learning, generative adversarial networks, and physics-informed networks, to speed up network convergence and relieve data reliance^26–28^.

Despite certain achievements, a major impediment persistently exists; datasets and networks are only disposable. That is, for each new task, all datasets and networks must be discarded, and new datasets and networks must be reconstructed. To achieve the unified design of various metamaterials, multiple “expert” networks have to be trained in a one-by-one manner, each of which is bound to an individual metamaterial^2^. Another “brute-force” method is to train a single “generalist” network using a larger dataset that covers various metamaterials^3^. Either way, each metamaterial is physically separated, and the data utilization efficiency is very low. Therefore, it is highly desirable to exploit whether there are any physical connections or network correlations among various metamaterials that can robustly handle a broad range of metamaterials.

Inspired by object-oriented C++ programming, for the first time, we propose a knowledge-inherited paradigm to break the stereotype that neural networks only work for predefined and shape-bound metasurfaces. It offers a fresh perspective that neural networks can also inherit the knowledge from the “parent generation” and then become freely assembled to construct an “offspring” neural network; this process is similar to building a container-type house. As a demonstration, we consider seven “parent” metasurfaces and train their ‘parent’ neural networks for the inverse design with an accuracy of over 93.8%. For a given “offspring” metasurface, these “parent” metasurfaces can be freely assembled in physical space, corresponding to the synthesis of “parent” neural networks managed by an assembled neural network. Different from transfer learning, our “knowledge-inherited learning” is unique and exclusive to metasurfaces (more details are discussed in Supplementary Note 5). Due to the inimitable physical character of metasurfaces, our knowledge-inherited network is associated with the complex spatial information of structures, which can further inherit the knowledge from “parent” metasurfaces, and then freely assemble for “offspring” metasurfaces. In other words, the synthesis of networks in the virtual space is inseparably correlated to the metasurface assembly in physical space. We benchmark the universality of our approach by one aperiodic metasurface and three periodic stretchable origami metasurfaces; their accuracies are far beyond those of conventional neural networks. Furthermore, we propose and experimentally demonstrate an innovative technology for spaceborne antennas in satellite communication, driven by intelligent origami metasurfaces. Our work opens up a new horizon towards the metasurface inverse design, excavating the inheritance feature to recycle the pretrained knowledge and enormously cut down the design dimensionality.

## Results

### Inspiration and paradigm of the knowledge-inherited neural network

**Fig. 1:**
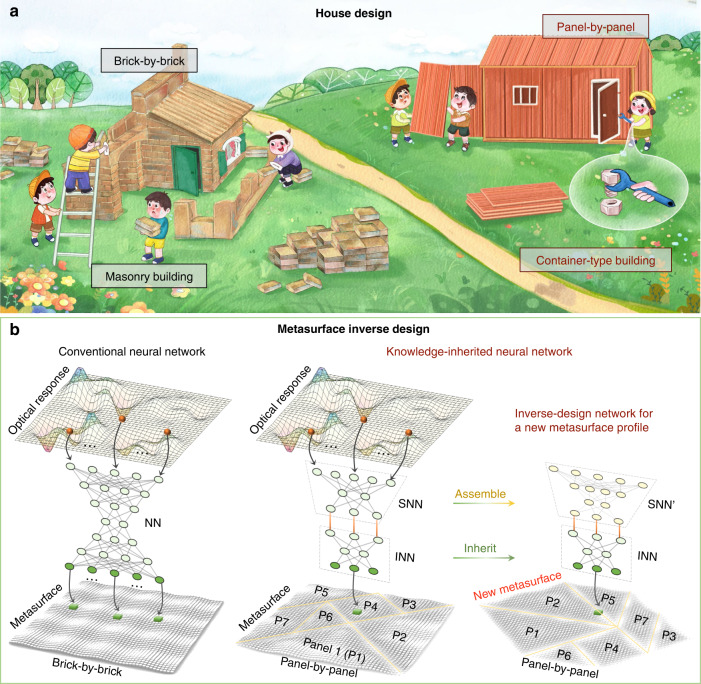
Schematic of the knowledge-inherited neural network for metasurface inverse design. **a** House building. In masonry buildings, all bricks are stacked and fixed with mortar, while container-type buildings are built with detachable “panel-by-panel” assemblies. **b** Knowledge-inherited paradigm for a metasurface inverse design. Similar to “brick-by-brick” masonry buildings, conventional neural networks are inseparable, fossilized, and single-functional once built up. In contrast, the proposed knowledge-inherited neural network is oriented for multi-object and shape-unbound metasurfaces. It is composed of two functional networks, the INN and the SNN. For a given “offspring” metasurface, we can synthesize the holistic neural network by assembling the INN and dynamically adjusting the SNN

The inverse design of metasurfaces has become a pervasive tool for numerous applications involving the direct generation of metasurface candidates for user-defined optical responses^29^. Such generation includes geometrical structures, material parameters, phase distributions, and spatiotemporal coding matrixs^17–20^. Existing machine learning works can be vividly described as the “brick-by-brick” paradigm because the input–output parameters of neural networks are predetermined and fixed. This is very similar to masonry buildings (Fig. 1a), where all bricks are stacked together and joined with mortar. Once built up, such a “brick-by-brick” masonry building is inseparable, fossilized, and single-functional.

An alternative container-type building has been found to be a profitable option due to the advantages of high flexibility, free assembly, recyclability, time savings, low cost, and labor savings. A clear-cut example is manifested in the rapid construction of numerous quarantine sites when human beings encounter the emergency public health crisis of COVID-19. It naturally raises the question of whether neural networks can also be freely assembled and recyclable. We term this novel network “panel-by-panel” or a knowledge-inherited neural network. Analogous to building a container-type house with high flexibility and free assembly, our method endows the network’s recyclability and flexible assemblability. It also breaks the stereotype that neural networks only work for predefined and shape-bound metasurfaces, which is similar to the fossilized masonry building. As schematically depicted in Fig. 1b, a knowledge-inherited neural network is composed of two functional networks, i.e., an inherited neural network (labeled INN) and an assembled neural network (labeled SNN). The INN is responsible for the inverse design of each “panel” metasurface, and the SNN functions as a deployer to assign the task for each INN. To clearly illustrate this point, we employ the lower right inset of Fig. 1b as an example. The database consists of seven “panel” metasurfaces, each of which has its own INN. For a given metasurface, such as a rectangle and a diamond shape, we first construct it with these seven “panel” metasurfaces in physical space, and then synthesize the holistic neural network by using the handy-prepared INN. In this procedure, the INN is completely inherited and reserved, and instead, we only need to dynamically adjust the SNN, enabling a green and data-efficient metasurface inverse design. For semantic description, we also term the “panel” metasurface the “parent” metasurface. Note that Fig. 1b is only a schematic of our network for metasurface inverse design. In the simulation and experiment, to verify the feasibility of our method handily, each small panel is set to be rectangular.

### Architecture of the knowledge-inherited neural network

**Fig. 2:**
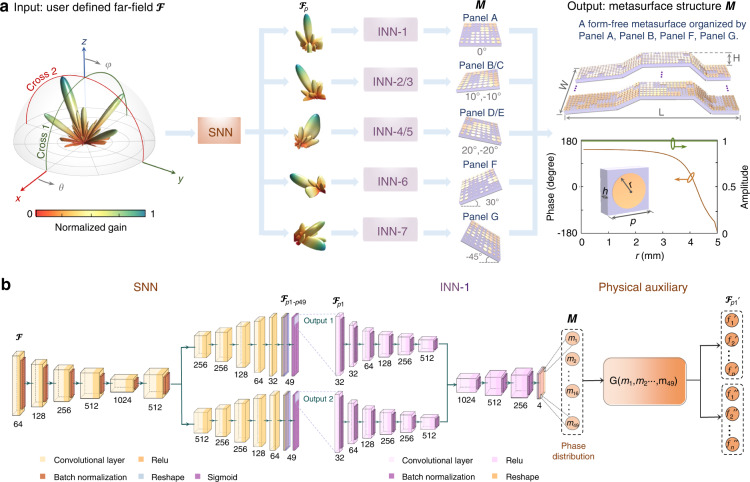
Design of the knowledge-inherited neural network. **a** Flowchart of the knowledge-inherited paradigm. To match the inheritance-to-assembly scheme, two networks (INN and SNN) are established, where the INN is responsible for the inverse design of each “panel” metasurface, and the SNN aims to explore the relationship between the global target EM response and the local EM response provided by each metasurface panel. For simplicity, we select two cross sections (*xoz*, *yoz*) of the RCS as input. We build up a data library that contains seven local panels with different tilt angles (tilt in the *φ* direction), including 0°, ± 10°, ± 20°, -30°, and 45°. The form-free aperiodic metasurface is composed of 49 local panels from Panels A, B, F, and G, with the whole size of *W* = 539 mm, *L* = 572 mm, and $H = 60{{{\mathrm{mm}}}}$. The geometrical details and dispersion relation of the meta-atom are presented on the right side, where $p = 10{{{\mathrm{mm}}}}$ and $h = 2{{{\mathrm{mm}}}}$. **b** The structure of the knowledge-inherited neural network. The SNN is a dual-output network comprised of a CNN, and the INN is established as a dual-input dual-output network with two modules, i.e., a CNN module for the inverse design and a physical auxiliary module for the forward mapping (Note that all digital subscripts represent the number of filters). They are concatenated by the intermediate phase distribution. More details are discussed in the Methods

Specifically, we establish a dataset containing seven “parent” metasurfaces with tilt angles of 0°, ± 10°, ± 20°, 30°, and -45° (see Fig. 2a and Supplementary Note 6 for details). Each “parent” metasurface has 8 × 8 unit cells and a phase response can cover 2π while the reflection amplitude remains almost in unity. The geometrical details and dispersion relations are displayed to the right of Fig. 2a. To alleviate the coupling effect among adjoint unit cells, we segment the “parent” metasurface into 4 × 4 super unit cells, i.e., each super unit cell has 2 × 2 uniform unit cells (Supplementary Note 3). Note that no matter which division strategy (that is, the size of the parent metasurface blocks) is all suitable for our method. In other words, our method has strong generality for any partition of the “offspring” metasurface (Supplementary Note 3).

INN is employed to comprehend the relationship from the complex far-field ${{{\mathcal{F}}}}_{P1}$ to the phase distribution *M* of the “parent” metasurface. The phase distribution *M* can be easily mapped into the practical metasurface according to the dispersion relation in Fig. 2a. Note that, although only a regular single circular patch is used in this work, other user-desired freeform style structure of meta-atoms (such as I-type, H-type, and so on) can also be applied. Accompanied by the complex spatial information, INN is established as a dual-input dual-output neural network, where both input and output are set as two channels (real and imaginary channels). Due to the existing nonuniqueness problem (namely, a nearly identical far field can be induced by multiple phase distributions)^30^, we attach a physical auxiliary module behind the INN. For the convolutional neural network (CNN) structured by the encoder-decoder model, two input channels are concatenated for fusing the processed features and further exporting the intermediate phase distribution *M* (Supplementary Note 1). For the physical auxiliary module, antenna theory^25^ is utilized to create the forward mapping from phase distribution *M* to the uniqueness and deterministic far-field ${{{\mathcal{F}}}}_{P1}^\prime$. To merge these two modules, the difference between the target far-field of each panel ${{{\mathcal{F}}}}_{P1}$ and the reconstructed far-field ${{{\mathcal{F}}}}_{P1}^\prime$ is taken as the loss function.

We then progress to the design of the SNN. Taking a large-scale form-free aperiodic metasurface (“offspring” metasurface) with a size of 539 × 572 × 60 mm^3^(56 × 56 unit cells) as an example, which is composed of 49 “panel”/“parent” metasurfaces with the combination of Panel A, Panel B, Panel F, and Panel G. Note that we use a more general principle based on structural periodicity to divide 3D metasurfaces here, rather than the periodicity of phase distribution. The SNN aims to decompose the holistic far field ${{{\mathcal{F}}}}$ to the far field contribution of each “panel” metasurface ${{{\mathcal{F}}}}_{P1\sim P49}$. For simplicity, we select two cross sections of far-field radar cross section (RCS), where the azimuth angles are 0° (cross 1) and 90° (cross 2). Conducted as a dual-output network, the SNN includes an encoder and two decoders, as shown in Fig. 2b. The dimension of the input is 4 × 91 × 2, corresponding to two channels. One channel (4 × 91 × 1) represents the frequency, which is presented by the same label, and the other channel represents the RCS, where 4 represents the number of azimuth angles (0° and 180°, 90°and 270°), and 91 represents the number of discrete points scanned over the elevation angle 0°–90°. The dimensions of the two outputs (one for the real part and the other for the imaginary part) are both 4 × 91 × 49, representing two cross sections of the RCS produced by the “offspring” metasurface. For these two outputs, the SNN is bifurcated in the decoder module, and two mean square error (MSE) loss functions are set, each possessing a weight of 0.5 (Supplementary Note 1). To further encapsulate the INN and the SNN, we unite the output of the SNN and the input of the INN in series. Specifically, the SNN outputs the far field of 49 “panel” metasurfaces, each of which is recommended with the corresponding INN of Panel A, Panel B, Panel F, and Panel G. The intermediate layer *M* of each INN is then extracted and synthesized into the ultimate metasurface arrangement.

### Results for aperiodic metasurfaces and a comparison with conventional neural networks

**Fig. 3:**
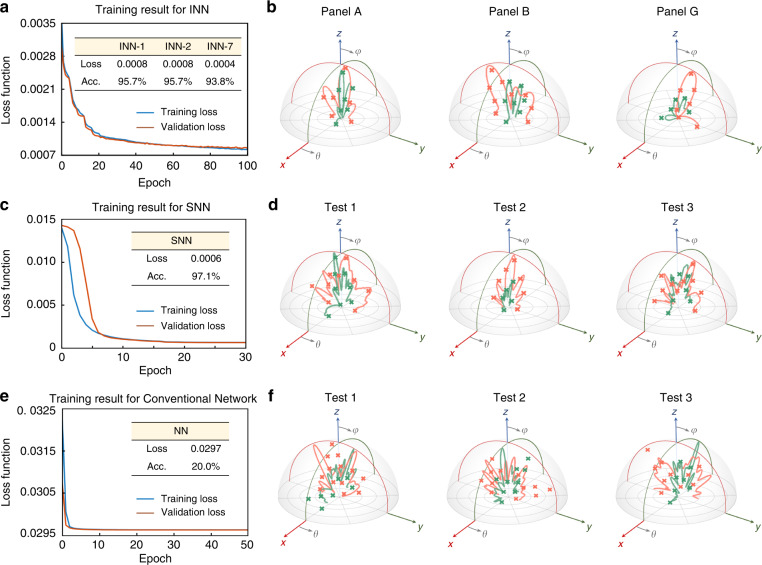
Training results and the comparison with a conventional neural network. **a** The training results for INN-1 over the epoch, with an accuracy of 95.7%. The accuracies for INN-2 and INN-7 are 95.7 and 93.8%, respectively. More results of the INN are left in Supplementary Note 2. The blue and red lines represent the training and validation losses, respectively. **b** The predicted RCS results of INN, where the three samples are selected from Panel A, Panel B, and Panel G, where the green color is for cross 1 and orange for cross 2. **c** The training results for the SNN over the epoch, with an accuracy of 97.1%. **d** Predicted RCS results of three samples by the knowledge inheritance paradigm. The predicted results are derived from the predicted meta-atoms. **e** The training results for the conventional neural network over the epoch. It is in a serious underfitting state, and the accuracy is only 20.0%. **f** Predicted RCS results of the three samples from the testing set

We trained the INN and the SNN with 50,000 and 15,000 samples, respectively, at frequencies of 8.0, 8.1, and 8.2 GHz (more details are discussed in Supplementary Note 2). The data are split into training, validation, and testing sets (80, 10, and 10%, respectively). Note that the testing set is isolated from the pretrained network, meaning that the data in a testing set has never been seen by the network. To intuitively characterize the accuracy of the knowledge-inherited neural network, we employ the Pearson correlation coefficient^31^, which is defined as follows:

1 $${\mathrm{accuracy}} = \frac{{{\mathrm{Cov}}(X,Y)}}{{\sqrt {{\mathrm{Var}}\left[ X \right]{\mathrm{Var}}[Y]} }} \times 100{{{\mathrm{\% }}}}$$

where *X* and *Y* represent the target RCS pattern and deep learning prediction, respectively. Figure 3a, c show the training results of the INN-1 and the SNN, where the testing accuracies reach 95.7% and 97.1%, respectively. The accuracies for the INN-2, INN-6, and INN-7 are 95.7, 94.3, and 93.8%, respectively (see Supplementary Note 2). Three samples of Panel A, Panel B, and Panel G are blindly picked in Fig. 3b. It is observed that each target RCS agrees well with that of the deep learning prediction for both cross 1 and cross 2. To further evaluate the performance of our knowledge-inherited paradigm, we expropriated terminal tandem accuracy (TT-accuracy), defined as the accuracy between the target pattern ${{{\mathcal{F}}}}$ and deep learning prediction induced by $\{ {{{\mathcal{F}}}}_{P1}^\prime ,{{{\mathcal{F}}}}_{P2}^\prime , \ldots ,{{{\mathcal{F}}}}_{P49}^\prime \}$. The TT-accuracy for this metasurface profile reaches 86.7%. Due to the cascade characteristics of our network, there may be a certain loss of the final accuracy. To further improve the accuracy of the network, there are also many ways, such as adjusting the network structure (number of layers, size of each layer, and activation function), parameter initialization, learning rate and batch size, or increasing the difference and randomness of the dataset. For the sake of intuition, we additionally extract the phase distribution *M* to calculate the predicted RCS by the knowledge inheritance paradigm, and three stochastic testing samples are selected (Fig. 3d). The ground truth and the predicted results are consistent with each other, providing unambiguous evidence for the reliability of our knowledge-inherited paradigm. Note that in our case, the far-field radiation is also only one example to prove the effectiveness of our network. More electromagnetic responses, such as transmission, band structure, and electric field, can also be extended.

As a comparison, we deal with the same design task with a conventional neural network. It is constructed by two modules: a CNN and a physical auxiliary module (Supplementary Note 4). We can see that, even with 50,000 groups of data at each frequency, the loss seems to be nonconvergent under a serious underfitting state, and the accuracy is only 20.0% (Fig. 3e). This is especially evident from the far-field comparison of the three test samples in Fig. 3f. The grave inconsistency of the results strongly indicates that the conventional neural network is completely invalid with the same training data. Further, we increased the complexity of this conventional neural network and found that there is no improvement in the accuracy (Supplementary Note 4). This comparison further illustrates the superiority and effectiveness of our strategy compared with other “disposable” design techniques.

### Knowledge-inherited neural network for periodic origami metasurfaces

**Fig. 4:**
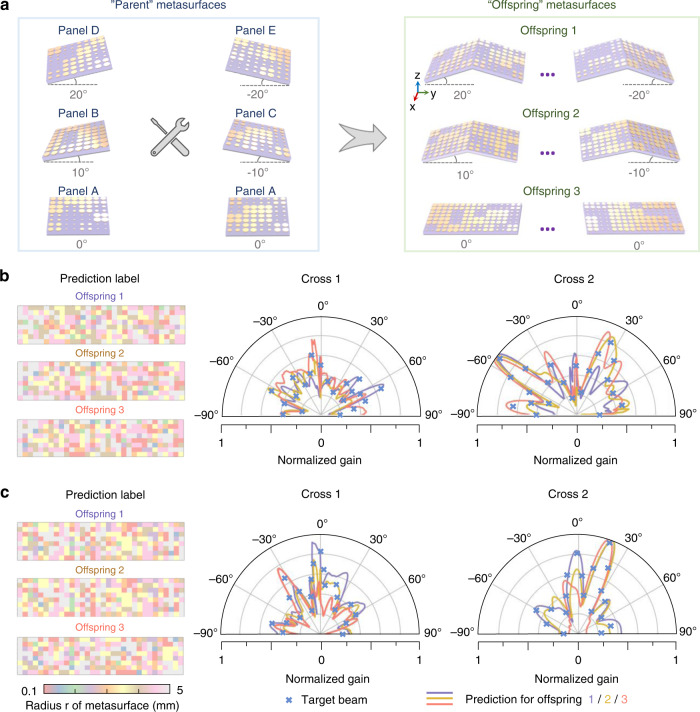
Demonstration of the knowledge-inherited paradigm for periodic origami metasurfaces. **a** Assembly process of periodic origami metasurfaces. Three “offspring” metasurfaces with separate stretchable angles (± 20°, ± 10°, and 0°) are organized by Panel D/E, Panel B/C, and Panel A, abbreviated as offspring 1/2/3. Each “offspring” metasurface contains 16 panels (including 16 × 64 unit cells), with 8 extending along the *y*-axis and 2 extending along the *x-*axis. **b**, **c** Prediction labels and RCS results of three “offspring” metasurfaces. Given the same specified far-field input (the blue cross), our knowledge-inherited paradigm for offspring 1/2/3 is capable of yielding similar on-demand scattering characteristics. The prediction labels, i.e., the prediction arrangement of metasurfaces, are presented on the left

To further benchmark the generalization of our novel design strategy, three periodic origami metasurfaces are established with separate stretchable angles of ±20°, ±10°, and 0°, termed offspring metasurfaces 1/2/3 (Fig. 4a). Organized by Panel D/E, Panel B/C, and Panel A, each offspring metasurface contains 16 “parent” metasurfaces (16 × 64 unit cells). For these new offspring, we only train the SNNs with 15,000 samples at frequencies of 8.0, 8.1, and 8.2 GHz. After hyperparameter optimization, these three offspring achieve satisfactory training results (Supplementary Note 2). The dimension of the input is 4 × 91 × 2, and that of the dual output is 4 × 91 × 16 (one for the real part and the other for the imaginary part). With one input (the user-defined far-field ${{{\mathcal{F}}}}$), the SNN is bifurcated in the decoder module here for two outputs and followed by two loss functions (Supplementary Note 1). Figure S2 presents the training results of each SNN, where the testing accuracies are 99.7, 97.4, and 98.4%, respectively. After reassembly with the corresponding INN, the TT accuracies of the three newly synthesized neural networks reach 94.5, 89.0, and 90.9%, respectively. Moreover, by feeding the same input into the three synthesized neural networks, the three offspring metasurfaces can yield similar scattering characteristics, as shown in Fig. 4b, c. The corresponding intermediate outputs of the phase distribution *M* are also presented. This remarkable performance means that by applying the simple inheritance-to-assembly scheme, we can not only robustly deal with a broad range of metasurface designs, but also derive versatile functions for many potential applications, such as satellite communication.

### Experimental results and the application in satellite communication

**Fig. 5:**
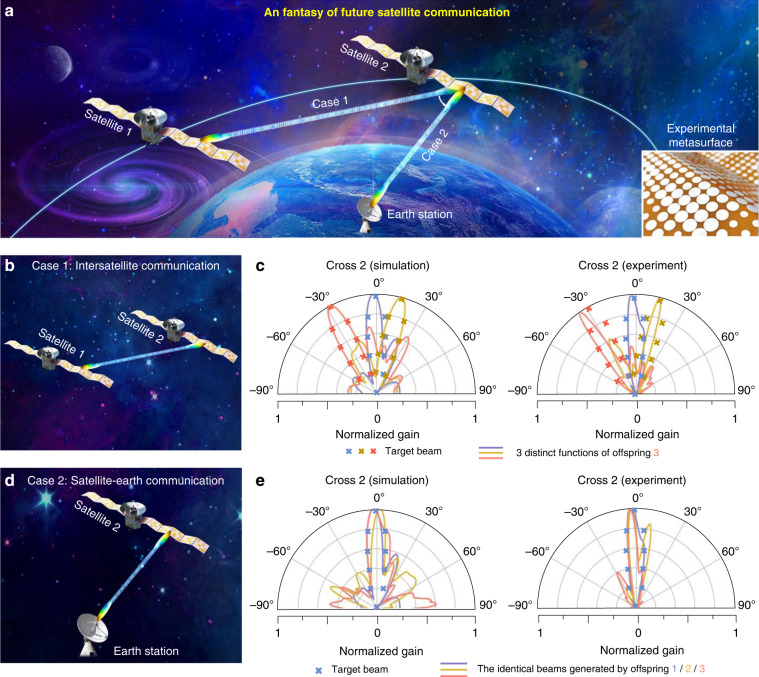
A fantasy of future satellite communication and experiments for origami metasurfaces. **a** Schematic of satellite communication based on intelligent origami metasurfaces. As a flexible, ultrathin, and low-cost competitor for free beam forming, the origami metasurfaces can be confirmed on the wings of satellites, which can be freely folded and stretched. Two satellites and one earth station are depicted to illustrate **b** intersatellite communication (case 1) and **d** satellite-earth communication (case 2). **c** Simulation and experimental results for case 1. For example, for offspring 3 in Fig. 4a, we show that distinct functions can be easily achieved by our knowledge-inherited paradigm. Conducted as the main section, only cross 2 is presented. **e** Simulation and experimental results for case 2. Considering that the satellites and receivers on Earth are relatively static, satellites with an offspring of 1/2/3 are able to generate identical beams

We emphasize that the flexible metasurface inverse design of periodic stretchable origami metasurfaces holds great potential in the application of satellite communication. Satellite communication has recently attracted growing interest due to the capability of global internet coverage, precise localization, and navigation. However, improving the satellite communication quality with lightweight and compatible communication facilities has been a major challenge. Serving as the “loudspeaker” of satellites, a spaceborne antenna is an indispensable part of satellite communication. The traditional spaceborne antenna technique has a considerably high hardware cost, energy consumption, and computational complexity^32^. Even with high accuracy, for inverse design, conventional electromagnetic solver usually depends on complicated numerical simulations, which necessitates iterative and lengthy calculations in a trial-and-error manner. In this respect, machine learning is heralded as a promising trade-off method to equipoise accuracy and computational efficiency in satellite communication. Consequently, we speculatively envision that intelligent origami metasurfaces can be formed (freely folded and stretched) on the wings of satellites, as schematically delineated in Fig. 5a. Not only is the volume greatly reduced before launching, but the antenna can also be intactly expanded and form a very large reflector after going into orbit. Thus, it provides great possibilities for the originality and forward-looking design of satellite communication.

Two typical communication situations are considered, i.e., intersatellite communication (case 1) and satellite-earth communication (case 2). For case 1, along the curved orbit, satellites require different beamforming to transmit signals between each other (Fig. 5b). Here, we employ offspring 3 in Fig. 4a as an example to show that distinct functions can be cushily achieved by our knowledge-inherited paradigm with the identical metasurface configuration. Conducted as the main section, the predicted and experimental patterns of cross 2 are shown in Fig. 5c, which are in accordance with three targets, especially for the main lobe. To further quantify the experimental efficiency, we define it as the efficiency ratio (the ratio of the predicted pattern to the target pattern) of the energy at full width at half maxima (FWHM):

2 $${\mathrm{efficiency}} = \frac{{E_{{\mathrm{FWHM}}}^{{\mathrm{Pre}}}/E_{{\mathrm{All}}}^{{\mathrm{Pre}}}}}{{E_{{\mathrm{FWHM}}}^{{\mathrm{Tar}}}/E_{{\mathrm{All}}}^{{\mathrm{Tar}}}}} \times 100{{{\mathrm{\% }}}}$$

where $E_{{\mathrm{FWHM}}}^{{\mathrm{Pre}}}$ ($E_{{\mathrm{FWHM}}}^{{\mathrm{Tar}}}$) is the energy at FWHM and $E_{{\mathrm{All}}}^{{\mathrm{Pre}}}$ ($E_{{\mathrm{All}}}^{{\mathrm{Tar}}}$) is the whole energy of the prediction pattern (target pattern). Therefore, the experimental efficiencies for three distinct functions in Fig. 5c are 99, 94, and 85%, respectively. For case 2, the satellite and the receiver on Earth are relatively static. Hence, satellite-earth communication requires unique vertical transmitting (receiving) beams (Fig. 5d). For example, satellites with offspring 1/2/3 can generate the identical beam, where the predicted and experimental RCS are shown in Fig. 5e, whose experimental efficiencies are 99, 92, and 95%, respectively. Note that the experiments are conducted at 8.0 GHz. The consistent results may be conducive to future satellite communication and other adaptive metadevices; see Supplementary Movie S1.

## Discussion

In conclusion, to the best of our knowledge, we, for the first time, have proposed a knowledge-inherited paradigm, also termed “panel-by-panel”, to be competent for object-oriented, large-scale, and shape-unset metasurface design. Endowed with an inheritance-to-assembly mixture scheme, the network can inherit the knowledge from the “parent” metasurface, and then disseminate knowledge for the reassembled “offspring” metasurface. It is the high flexibility and free assemblability of this scheme that endows our method with high generality. Such green and correlation metasurface design is different from conventional neural networks that are disposable and inseparable. In other words, the knowledge-inherited network considerably enhances the adaptability and exponentially scales down the dimensionality; this is very obvious from the comparison in Fig. 3e. In practice, by applying our inheritance-to-assembly strategy, we can also achieve multi-stage network assembly for larger or more complex models, such as an irregular bird-like metasurface (Supplementary Note 7). Another point we would like to emphasize is that the advantages of “panel-by-panel” are extremely maximized in the application of geometrical periodic metasurfaces, such as origami metasurfaces. Attached with external force stimulation, origami structures can smoothly dominate their folding/unfolding movement to form a “modular” metasurface with excellent structural stiffness and adjustable periodicity, which firmly coincides with our “panel-by-panel” policy. Its compatibility and lightweight characteristics also appeal to underlying applications in satellite communications. As a high coverage communication system, satellite communication can flexibly peruse multiple access communication and channel on-demand allocation, offering terrific signals for every corner of the world, even in remote mountainous areas or Mount Everest^32^. Further, the design principles can be generalized to higher frequencies and broadband by exploring novel metasurface structures and unorthodox networks. Combined with complex-amplitude metasurfaces, it is also promising to customize more kinds of scattering patterns for complicated application scenarios.

Furthermore, we expect that the concept of the knowledge-inherited network demonstrated here could be deployed in many application areas where machine learning is used, with compelling applications throughout, including plasmonic structures, photonic circuits, imaging recognition, biomedicine, and quantum computing^33–38^. For example, thus far, optical technologies have mainly used photonic optimization in a restricted design space, largely limiting the structural topology/geometry and shape^3^. Associating with our generic and hierarchical network strategy may inject new prospects for this challenge, and the pipeline is as follows: first, we establish a “bank” that contains a glut of well-trained ‘parent’ networks for diverse restricted spaces, then attached to a “deployer” network for seeking the potential interaction. Such a novel perspective indisputably offers a fresh mentality for machine learning, further alleviating its tension with big data and high-performance central processing units (CPUs).

## Materials and methods

### Data generation

The far-field cross-section data of different metasurfaces is generated through antenna theory, where each meta-atom/unit cell is regarded as an independent radiation source, and the far-field is calculated by accumulating the contributions of all meta-atoms. In doing so, we can significantly save computational time for data collection. To reduce the coupling effect among adjacent meta-atoms, we deploy a super meta-atom containing 2 × 2 equal-sized units with the dimension of *D*. Under the normal incidence of plane waves, the far-field function scattered by the metasurface is expressed as follows:

3 $$f\left( {\theta ,\varphi } \right) = \mathop {\sum }\limits_{m = 1}^M \mathop {\sum }\limits_{n = 1}^N U_{mn}{{{\mathrm{exp}}}}\{ ikD[\left( {m - 1} \right)sin\theta cos\varphi + \left( {n - 1} \right)sin\theta sin\varphi ]\}$$

where *θ* and *φ* are the elevation and azimuth angles of an arbitrary direction, respectively, and *U*_*mn*_ is the complex voltage of the unit cell located at (*m*, *n*). *M*(*N*) is the number of unit cells along the *x*(*y*) axis, and *m*(*n*) represents the *m*-th(*n*-th) unit cell inside. *k* represents the wavenumber. For tilted “panel” metasurfaces, assuming the panel rotates at an angle of *α* in the −*φ* direction, as shown in Fig. S10, the modified far-field function is expressed as follows:

4 $$f\left( {u,v} \right) = \mathop {\sum }\limits_{m = 1}^M \mathop {\sum }\limits_{n = 1}^N U_{mn}{{{\mathrm{exp}}}}\{ ikD[\left( {m - 1} \right)u + \left( {n - 1} \right)vcos\alpha - (n - 1)\sqrt {1 - u^2 - v^2} sin\alpha ]\}$$

where $$u = sin\theta cos\varphi$$, $$v = sin\theta sin\varphi$$.

In this way, we first generate 50,000 datasets of each “parent” metasurface by randomly setting the metasurface distribution for INN training. Then, for an “offspring” metasurface (assembled by “parent” metasurfaces), we also follow a similar procedure to generate 15,000 samples for SNN training.

### Deep learning architecture

The INN is established as a dual-input dual-output network that is divided into two modules, i.e., a CNN module for the inverse design and a physical auxiliary module for the forward mapping. For the CNN module, there are 13 convolution layers and two deconvolution layers, in which the seventh layer of the two input channels are concatenated for fusing the processed features and further exporting the intermediate phase distribution *M*. For the physical auxiliary module, antenna theory is utilized to unfold the forward mapping and further outputs both the real and imaginary parts of the far field. Conducted as a dual-output network, the SNN for the proposed form-free aperiodic metasurface consists of two parts, an encoder and a decoder, by using five convolution and 11 deconvolution layers, which are bifurcated in the second deconvolution layer, as shown in Fig. 2b. The detailed structure and operating process of our knowledge-inherited paradigm (INN and SNN, respectively) are provided in Supplementary Note 1. All models are built under a CPU of Intel (R) Core (TM) i7-8700K and a graphics processing unit (GPU) of NVIDIA GeForce RTX 2080 SUPER.

### Experimental measurement

The experiment was carried out in an anechoic chamber, mainly including a transmitting horn antenna and a receiving horn antenna. In the measurement, the origami metasurfaces and the transmitting horn antenna were fixed on a rotating platform at a distance of 1 m, and the receiving horn antenna was placed 8 m away from the rotating platform. Furthermore, the rotating platform was digitally controlled to rotate within −90° to 90°. The receiving horn antenna was connected to a vector network analyzer (VNA) to detect the scattered field, including the amplitude and the phase information. In addition, when the rotating platform is turned to 0°, the transmitting antenna, the receiving antenna, and the central point of the scatterer were kept in the same vertical plane.

## Supplementary information

Supplementary Information for A knowledge-inherited learning for intelligent metasurface design and assembly

Supplementary Movie

## Data Availability

The data that support the findings of this study are available from the authors on reasonable request.
