# Supplementary material for: A knowledge-inherited learning for intelligent metasurface design and assembly
Source: Light Sci Appl. 2023 Mar 30;12:82. doi: 10.1038/s41377-023-01131-4 (PMC10060944; doi:10.1038/s41377-023-01131-4)
Supplement: Supplementary file 1 — Supplementary Information for A knowledge-inherited learning for intelligent metasurface design and assembly [file 41377_2023_1131_MOESM1_ESM.docx]

Supplementary Information for

**A knowledge-inherited learning for intelligent metasurface design and assembly**

Yuetian Jia^1,2,3^, Chao Qian^1,2,3^*^,^*^*^, Zhixiang Fan^1,2,3^, Tong Cai^1,2,3,4^, Er-Ping Li ^1,2,3^ and Hongsheng Chen^1,2,3,*^

*^1^ ZJU-UIUC Institute, Interdisciplinary Center for Quantum Information, State Key Laboratory of Extreme Photonics and Instrumentation, Zhejiang University, Hangzhou 310027, China.*

*^2^ZJU-Hangzhou Global Science and Technology Innovation Center, Key Lab. of Advanced Micro/Nano Electronic Devices & Smart Systems of Zhejiang, Zhejiang University, Hangzhou 310027, China.*

*^3^ Jinhua Institute of Zhejiang University, Zhejiang University, Jinhua 321099, China.*

*^4^Air and Missile Defense College, Air Force Engineering University, Xi' an, 710051, China.*

*^*^Corresponding authors: chaoqianzju@zju.edu.cn* *(C. Qian);* [*hansomchen@zju.edu.cn*](mailto:hansomchen@zju.edu.cn) *(H. Chen)*

**The PDF file includes:**

- Supplementary Note 1: Structure and operating process of the knowledge-inherited paradigm
- Supplementary Note 2: Training results of the knowledge-inherited paradigm
- Supplementary Note 3: Division strategy of “parent” and “offspring” metasurfaces
- Supplementary Note 4: Structure of conventional neural network
- Supplementary Note 5: Comparison with transfer learning
- Supplementary Note 6: Evolutive antenna theory for tilted metasurfaces
- Supplementary Note 7: Another example for free-form metasurface design
- Supplementary Note 8: Experimental measurement and fabrication

**Other Supplementary Information for this manuscript includes:**

- Supplementary Movie S1 (.mp4 format): A movie about the conceptual introduction and wireless application of the knowledge-inherited neural network. At the first half of the movie, we activate the scenario in Fig. 5a of the main text to show a fantasy of future satellite communication enabled by intelligent origami metasurface. Then, taking gene recombination as a vivid metaphor, we show the dynamic synthesis of the neural network, where INN is analogous to gene fragment that can be inherited to “offspring” metasurface, and SNN connects each INN to synthesize a holistic neural network (like a fresh gene chain).

**Supplementary Note 1: Structure and operating process of the knowledge-inherited paradigm**

The proposed knowledge-inherited paradigm contains two specific networks, i.e., the INN and the SNN, with tangible missions. All models are built under a central processing unit (CPU), i.e., Intel (R) Core (TM) i7-8700K, and a graphics processing unit (GPU), i.e., NVIDIA GeForce RTX 2080 SUPER. Employing periodic origami metasurfaces as an example, we elaborate on the detailed structure and operating process of our knowledge-inherited paradigm, as shown in Fig. S1. The INN is responsible for the inverse design of each “panel” metasurface from the complex far-field $\mathcal{F}_{P1}$ to the phase distribution $M$, which is established by the CNN. For the CNN module, each convolutional and deconvolutional layer is followed by Batch Normalization and ReLU layers, which not only accelerates the training speed but also strengthens the robustness of the network and alleviates the vanishing and overfitting gradient problems^S1^. Accompanied by the physical auxiliary module, the INN is finally established as a dual-input dual-output neural network (one channel for the real part and one for the imaginary part). The dimensions of both inputs are 4 × 91 × 1, and that of the intermediate output of the CNN module is 4 × 4 × 4, representing the continuous phase distribution of each “panel” metasurface with 16 super unit cells. The two channels are then concatenated in the 7^th^ convolution layer to fuse the processed features and further export the intermediate phase distribution $M$. After going through the physical auxiliary module, the dimensions of the real and imaginary output are finally transformed into 4 × 91 × 1.

Functioning as a deployer, the SNN aims to explore the relationship between the global target EM response and the local EM response provided by a series of metasurface panels, which is established as a dual-output network. Composed of two parts, i.e., the encoder and the decoder parts, by applying 5 convolution and 11 deconvolution layers, the SNN is bifurcated in the 2^th^ deconvolution layer for the real and the imaginary output. All layers are attached with BN before bifurcating, while there is no BN after bifurcating. The dimension of the input is 4 × 91 × 2, which consists of two channels, and each is a 4 × 91 matrix (one channel represents the frequency, and the other channel represents RCS, where the elevation angles 0°$\sim$90° are discretized into 91 data points). The dimensions of the two outputs (one for the real part and another for the imaginary part) are both 4 × 91 × 16, representing two cross sections of the RCS produced by 16 “panel” metasurfaces. To further synthesize the INN and SNN, we connect the output of the SNN and the input of the INN in a series. In detail, the SNN outputs the far-field of 16 “panel” metasurfaces, each of which is imported into the corresponding INN of Panel D/E, Panel B/C and Panel A for offspring 1/2/3. Then, the intermediate layer $M$ of each INN is extracted and integrated into the ultimate metasurface arrangement prediction.

To merge the two modules in the INN, the difference between the target far-field of each panel $\mathcal{F}_{P1}$ and the reconstructed far-field ${\mathcal{F}_{P1}}^{'}$ is taken as the loss function, which is the same for both the real and the imaginary parts of far-field $\mathcal{F}_{P1}$. For the SNN, the difference between the ground truth and the prediction results of $\mathcal{F}_{P1\sim P16}$ (the real and the imaginary parts) is taken as the loss function. Here, we use the classical mean square error (MSE) loss function^31^ for the gradient descent algorithm^S2^. The MSE is defined as follows:

$MSE= \frac{1}{N}\sum_{i=1}^{N} {(f_{P\_i}-{f_{P\_i}}^{'})}^{2}$ (S1)

where $f_{P\_i}$(${f_{P\_i}}^{'}$) represents the $i_{th}$ sampling point of the ground-truth (predicted) far-field cross-section and $N$ represents the 4 × 91 discrete points of RCS. Since both SNN and INN are established with dual inputs and dual outputs, the independent loss function will be applied to each output, that is, the final loss is contributed by the summation of each sub-loss. Therefore, we assign different weights to each sub-loss to flexibly adjust its contribution to the total training loss, and the synthesized loss function is defined as follows:

$Loss= \alpha L_{re}+\beta L_{im}$ (S2)

where $L_{re}$($L_{im}$) represents the loss for the real (imaginary) output and $\alpha$($\beta$) is the corresponding loss weight. Since the real part and the imaginary part of optical response are equally important, we set both the weights ($\alpha$and$\beta$) as 0.5.


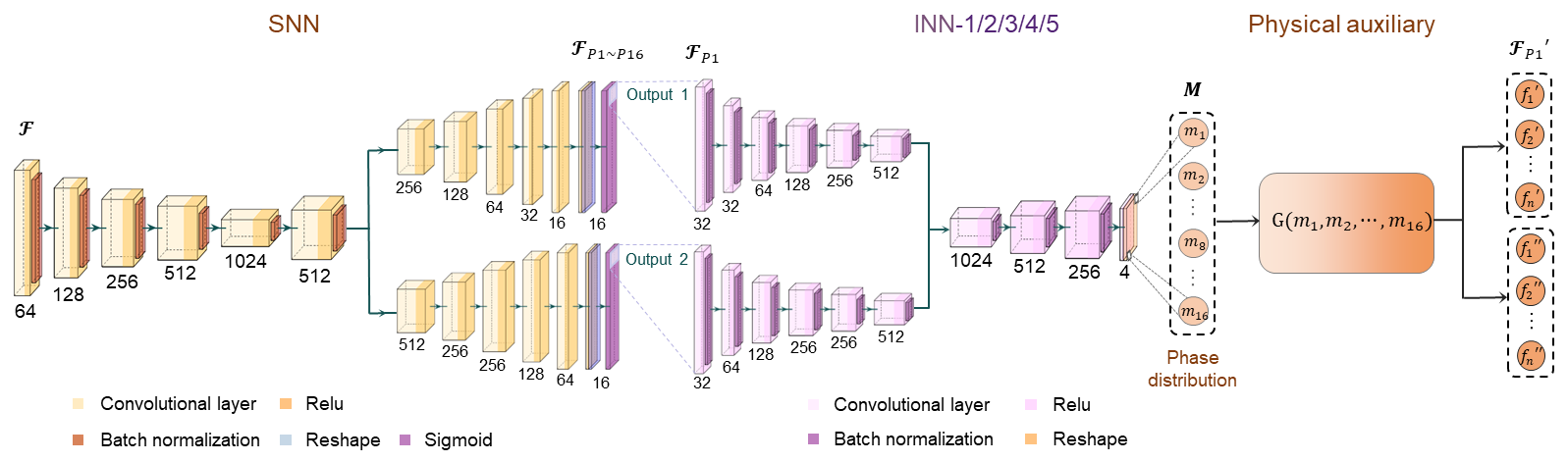


**Figure S1 | Detailed structure of the knowledge-inherited paradigm for periodic origami metasurface.** For the three periodic offspring, we only need to assemble different SNNs to build a brand new network for specific metasurface design. Here, the SNN is also a dual-output network which is comprised of a CNN.

**Supplementary Note 2: Training results of the knowledge-inherited paradigm**

We train the INN and SNN on 15,000 and 50,000 theoretical samples, respectively, at 8.0, 8.1, and 8.2 GHz, the results of which are randomly split into training, validation and testing sets ($80\%$, $10\%$and $10\%$, respectively). The Adam optimizer^S3^ is employed to update the parameters to complete the training of the models. During training, the epochs and batch size^S2^ of the INN are set to 100 and 64, and those of the SNN are set to 30 and 128, respectively. The initial learning rates of the INN and the SNN are 0.0001 and 0.001, respectively, which will continue to shrink by a ratio of 0.5 when the validation loss stops improving, and the update is terminated when the minimum learning rate of ${10}^{-5}$ is reached. In this way, the optimal model can be obtained quickly and accurately. The training results of the INN and SNN are all shown in Fig. S2, where the validation losses are consistent with the training losses and all of them are close to 0 with high accuracies over 93.8%. More results at different frequencies are shown in Fig. S3.


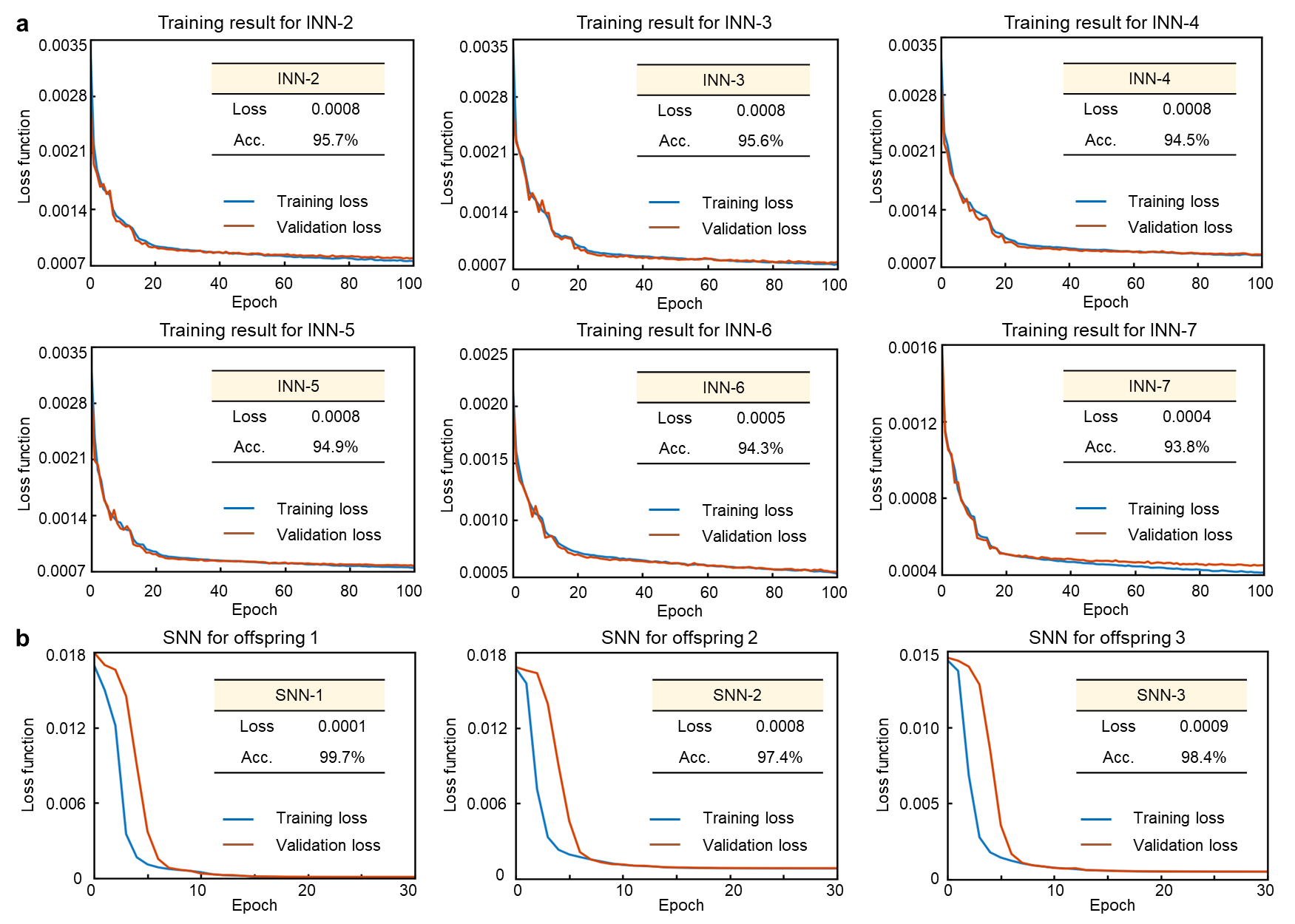


**Figure S2 | Training results** **of the knowledge-inherited paradigm.** Training results of (**a**) INN and (**b**) SNN for periodic origami metasurfaces. For both the INN and SNN, the validation losses are consistent with the training losses, and all of them are close to 0 with high accuracies over 93.8%. The blue and red lines represent the training and validation losses, respectively.


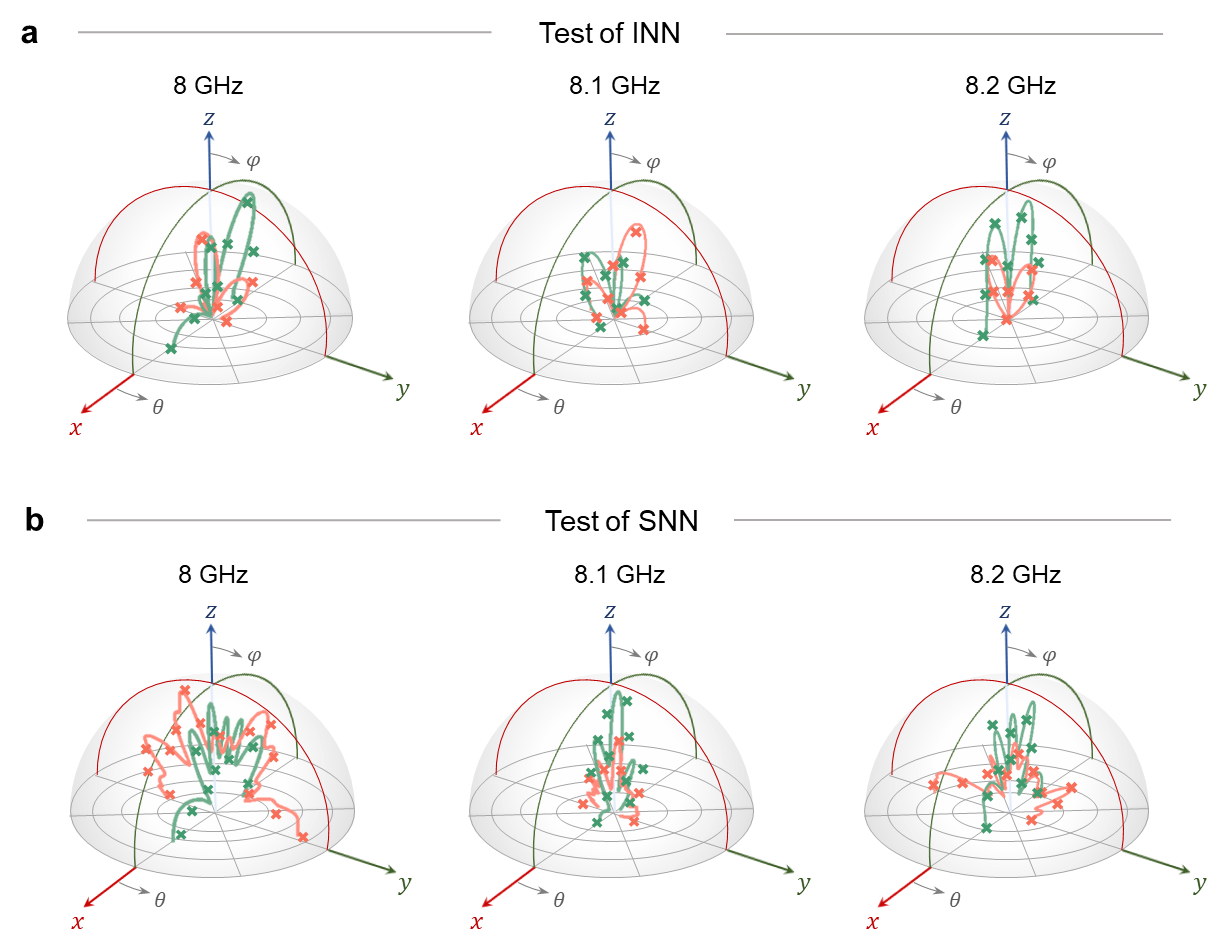


**Figure S3 | The testing samples of** (**a**) **INN and** (**b**) **SNN at different frequencies.**

Further, we carry out experiment to observe the accuracy limit with respect to the amount of data. Taking the periodic metasurface (offspring 3) in Fig. 4 as an example, Fig. S4 represents the variation trend of accuracies of INN、SNN and the knowledge-inherited paradigm, which rises rapidly with the increase of data and then tends to be stable. Considering both the time of data collection and the accuracy of networks, we finally determined the training data for INN and SNN as 50,000 and 15,000. It also shows that the upper limit of the accuracy of this synthesized network is about 91.2%.


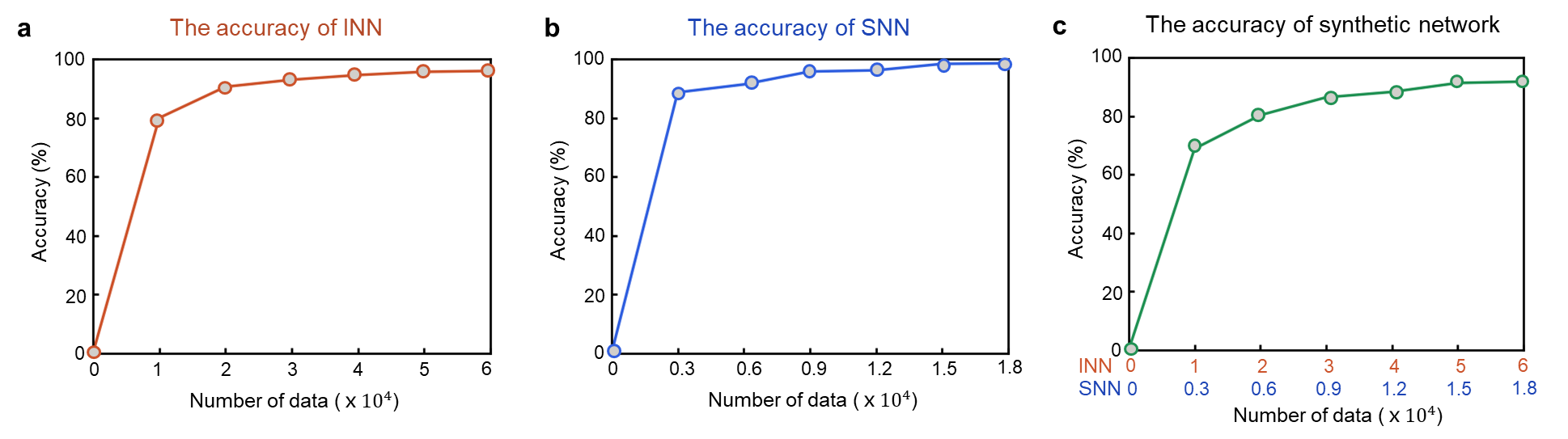


**Figure S4** **|** **The accuracies of** (**a**)**INN,** (**b**)**SNN and** (**c**)**the synthetic network with respect to the increase of data.**

**Supplementary Note 3: Division strategy of “parent” and “offspring” metasurfaces**

For the division strategy of “parent” metasurfaces, we deploy super meta-atom (containing 2 × 2 equal-sized meta-atoms) treatment to reduce the coupling effect among adjacent meta-atoms. To prove the effectiveness of super meta-atom, we take three metasurfaces as examples and perform simulation (by CST) and theoretical (by antenna theory) calculation. It is obvious from Fig. S5 that the simulation and theoretical results are similar when using super meta-atom technique, in stark contrast to the results without using super meta-atom.


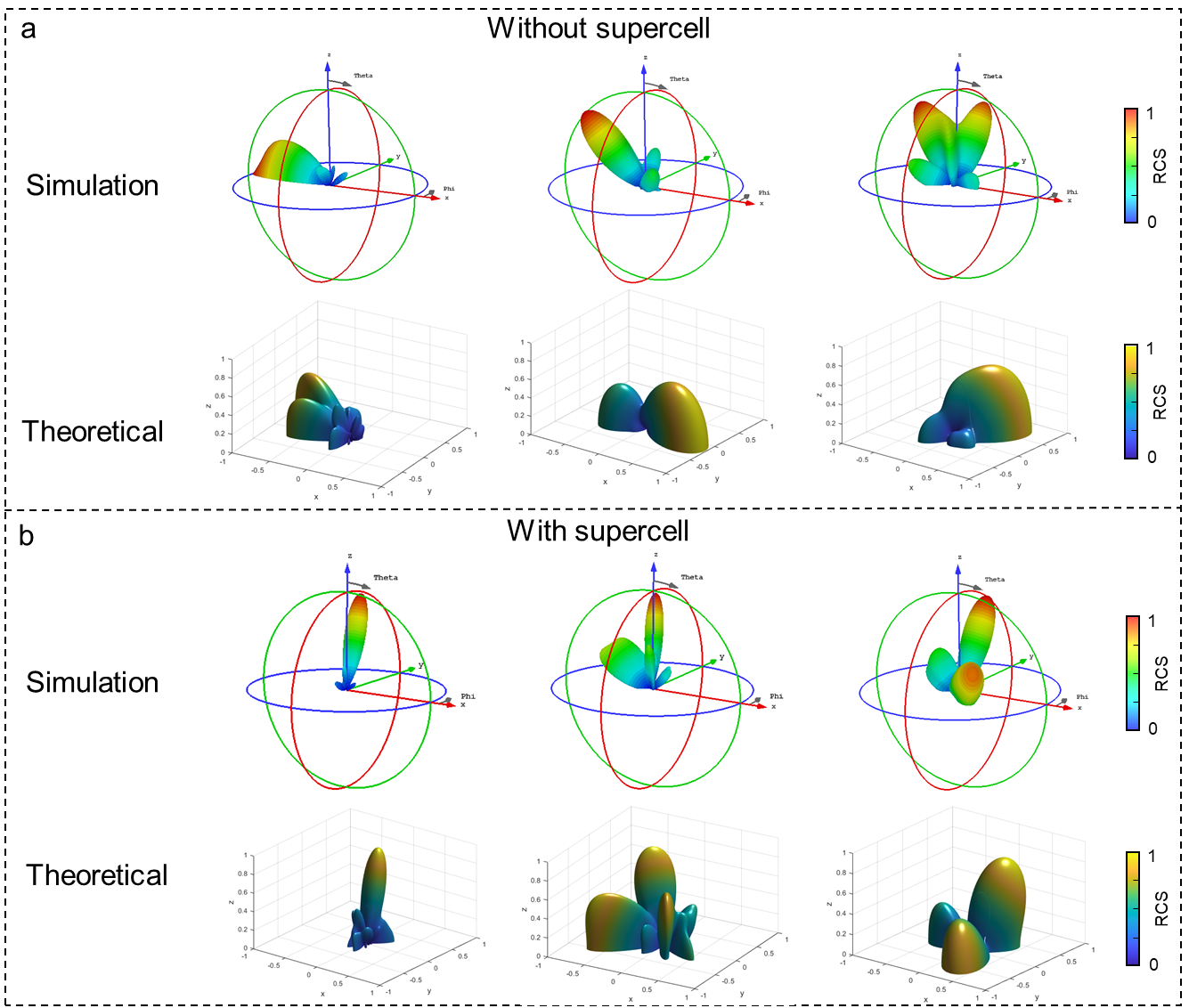


**Figure S5 | Result comparison between** (**a**) **without** **and** (**b**) **with super meta-atom technique.**

For the division strategy of “offspring” metasurfaces (that is, the size of the parent metasurface blocks), we want to clarify that other division strategy is also suitable for our method. In other words, our method has strong generality for any partition of the “offspring” metasurface. For the same “offspring” metasurface, as long as the INNs corresponding to the assembled “parent” metasurfaces and a suitable SNN are well trained, we can easily realize the metasurface design by our inheritance-to-assembly mixture scheme. To certify this point, we make another division of the form-free metasurface in Fig. 2a of the manuscript. As shown in Fig. S6a, we construct another four “parent” metasurfaces (Panel A’/B’/C’/D’) each of which contains 8 × 16 units. The training results for these four panels (INN 1’/2’/3’/4’) with high accuracies are shown in Fig. S6a. Note that for the convenience of division, we reduced the size of the “offspring” metasurface in Fig. 2a to 539 × 572 × 60 ${mm}^{3}$ (56 × 48 unit cells), which can be organized by 21 “parent” metasurfaces. Hence, for the new SNN’, the dimension of the input is 4 × 91 × 2 and that of the output is 4 × 91 × 21. The training result of SNN’ with an accuracy of 98.7% (Fig. S6b) proves that it functions as an excellent deployer which can assign accurate task for each INN. The terminal tandem accuracy of this newly synthesized network reaches 85.3%, further demonstrating the universality of our strategy.


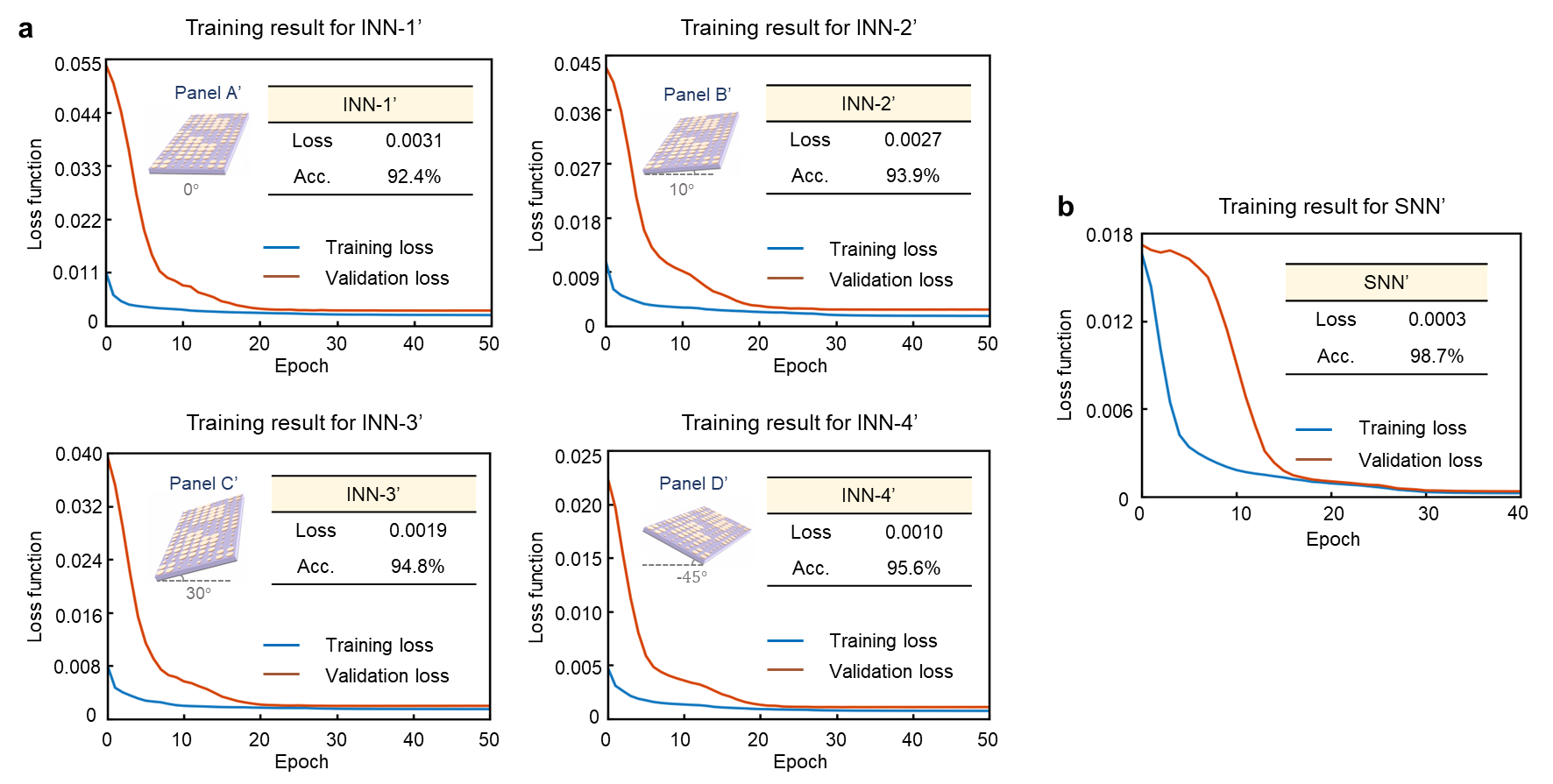


**Figure S6 | Training results for new** (**a**) **INN’ and** (**b**) **SNN’ with high accuracies.**

Further, we conduct some simulations to observe the mutual interference between panels that may affect the design accuracy. We implement other two arbitrary assemblies of four picked panels and perform theoretical and simulation calculation (Figs. S7a and S7b). The 2D far-field turns out that the theoretical results are also consistent with the simulation. It further proves that the mutual interference of each panel has little effect on the accuracy. Probably, an ulteriorly optimization on metasurface structure can also suppress the coupling effect. For example, adding metal rim on the top of meta-atoms. As shown in Fig. S7c, we apply meta-atoms with metal frames in the 8 × 8 planar metasurface, whose 2D far-field also turns out that the theoretical results are generally consistent with the simulation. It demonstrates that coupling suppression can be achieved through some ingenious structural design, whose effect is similar to that of our method. More importantly, we note that more or less coupling effect actually does not influence the main contribution of our paper––the novelty of the knowledge-inherited paradigm.


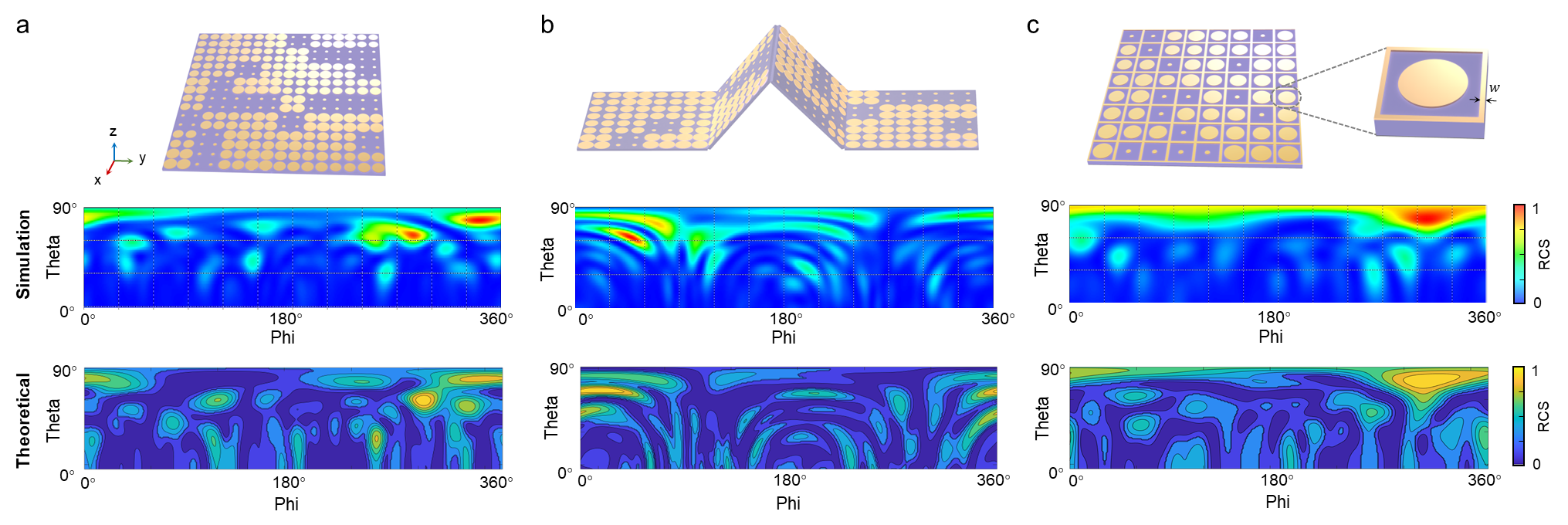


**Figure S7 | Comparisons between theoretical and simulation results of three metasurfaces.** (**a**, **b**) Two metasurfaces are arbitrarily assembled by four picked panels, whose 2D form of the far-field results are presented. (**c**) Another example of 8 × 8 planar metasurface with meta-atoms with metal frames (w=0.5 mm) are presented to prove the successful coupling suppression.

**Supplementary Note 4: Structure of** **conventional neural network**

As a comparison, we also deal with the same design task with conventional neural network. It is constructed by two modules: a CNN and a physical auxiliary module. The CNN module consists of two parts, an encoder and a decoder, created by using eight convolutional and two deconvolutional layers, as shown in Fig. S8. Each convolutional and deconvolutional layer is followed by BN and ReLU. For the encoder part, the radiation pattern matrix is transformed down from 4 × 91 × 1 to 1 × 1 × 1024 to extract the features from the far-field RCS. For the decoder part, the matrix is transformed from 1 × 1 × 1024 to 28 × 28 × 4 to map the image from a small resolution to the specific large-resolution output. For the physical auxiliary module, the output of the phase distribution is fed into the antenna theory to obtain the target far-field. The MSE difference between the ground truth and the prediction result of the far-field is taken as the loss function.

**
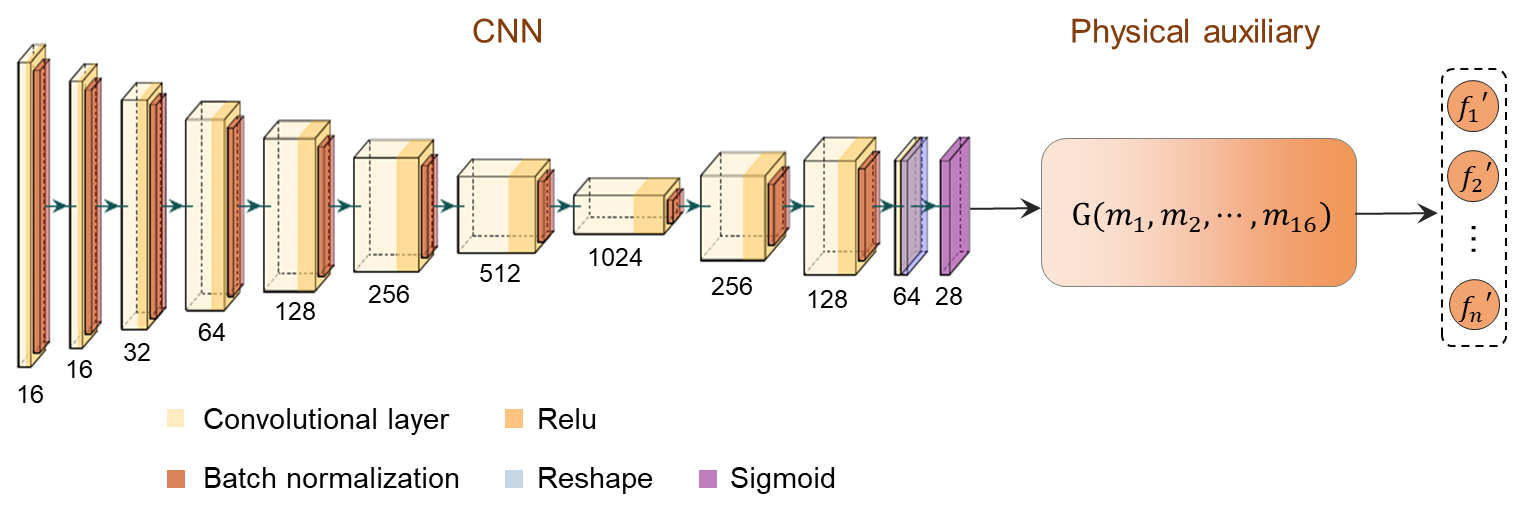
**

**Figure S8 | Structure of conventional neural network.** The conventional neural network is constructed by two modules: the CNN and the physical auxiliary module. They are concatenated by the intermediate phase distribution.

Further, we increased the number of layers of the conventional neural network to 12 and 20. However, as shown in Fig. S9, we do not see any improvement on the accuracy. We analyze the underlying reason is that, for large-scale metasurface design task, the solution space is so enormous that the existing data volume is far from enough to train the conventional neural network, so it is useless to increase the number of network layers. This further confirms the high performance of our method.


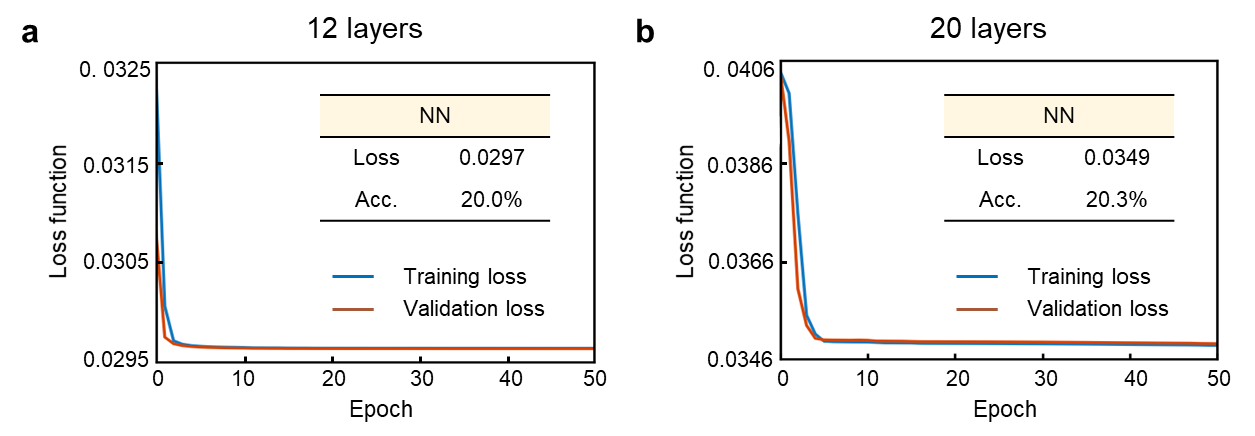


**Figure S9 | Training results for conventional network with** (**a**) **12 layers and** (**b**) **20 layers.**

**Supplementary Note 5: Comparison with transfer learning**

At the first glance, our method and transfer learning may seem similar. However, we would like to particularly emphasize that the underlying mechanism and performance are fundamentally different. And our method has not been mentioned in any previous study and cannot be readily duplicated from/to computer science. Their differences are explained below, including operation principle and performance comparison.

Transfer learning is a mature algorithm extended from computer science. The basic operation process is to transfer the pre-trained neural network in the source task to assist the training of target task. However, the performance of transfer learning cannot be guaranteed. Sometimes, the performance even becomes worse compared with that without transfer learning. In other word, transfer learning is like a ‘black box’ without revealing its internal mechanism, which heavily relies on the brute-force attack of features and lacks reasonable explanation (Fig. S10a).

By contrast, our “knowledge-inherited learning” is a unique and exclusive method, which can be regarded as a ‘white box’ with physical connection between the internal transferred knowledge (Fig. S10b). This is the first time to propose this method and it is not extended from computer science. Due to the inimitable physical character of metasurfaces, our knowledge-inherited network is associated with the complex spatial information of structures, which can further inherit the knowledge from “parent” metasurfaces, and then freely assemble for “offspring” metasurfaces. The specific process is shown in the Fig. S11. In other words, the synthesis of networks in the virtual space is inseparably correlation to the metasurface assembly in physical space. Further, combined with a physical auxiliary module in each INN, we can obtain the phase distribution of each assembled panel easily and accurately without the non-uniqueness issue (namely, a nearly identical far field can be induced by multiple phase distributions).


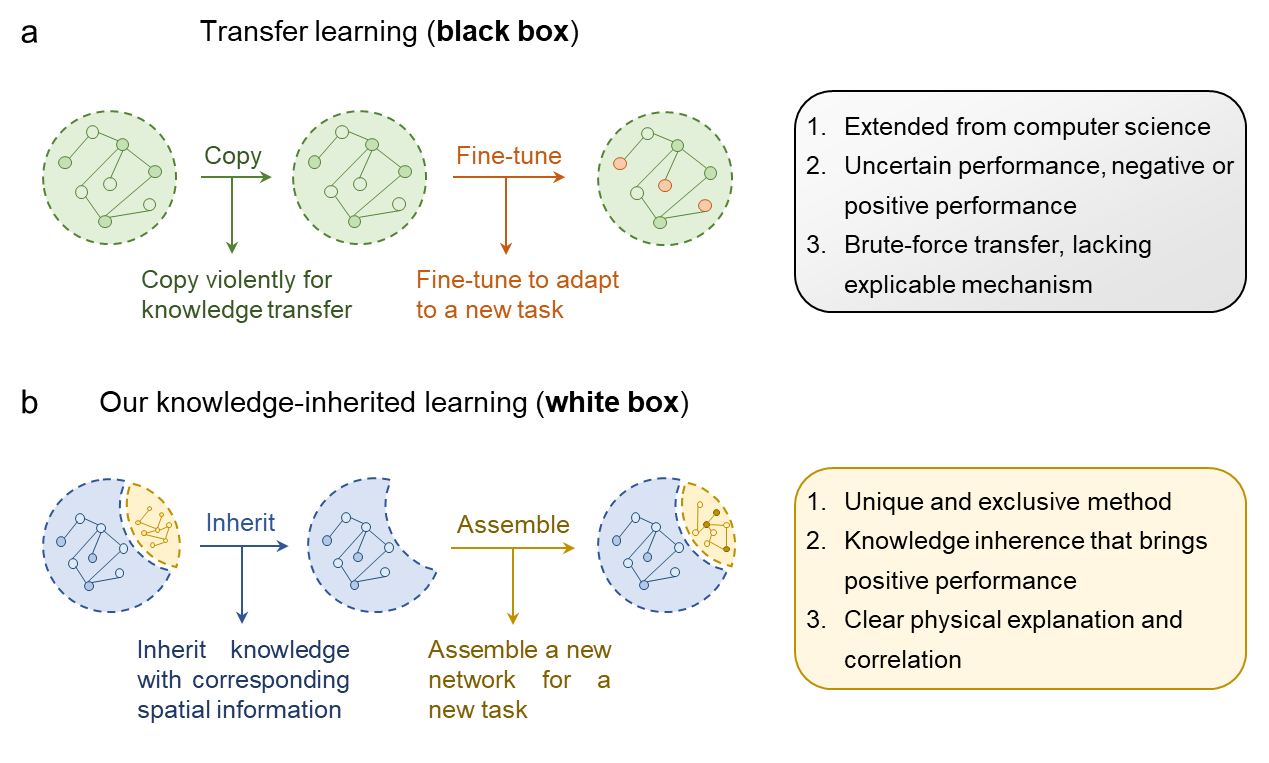


**Figure S10 | Comparison between** (**a**) **transfer learning and** (**b**) **our knowledge-inherited learning.**


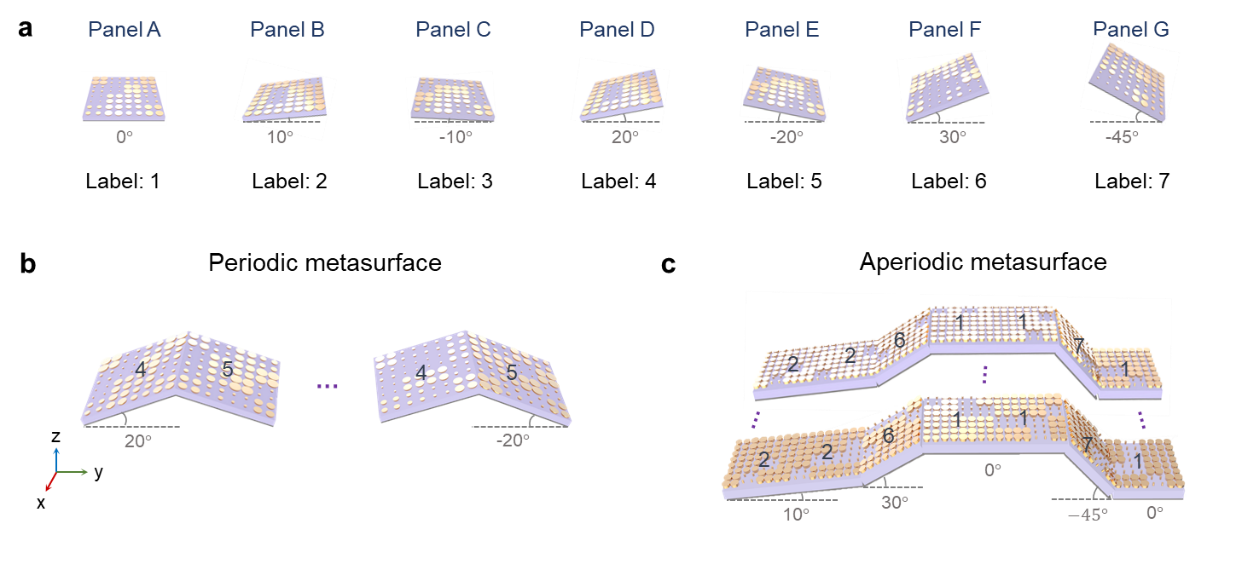


**Figure S11 | ‘Parent’ metasurfaces are physically assembled in sequence to form different ‘offspring’ metasurfaces.** The numbers on (**b**, **c**) two ‘offspring’ metasurfaces represent the label of different (**a**) ‘parent’ metasurfaces.

From the perspective of performance, for transfer learning, it only works when the features of the depth model in the first task are generalization features. However, in our knowledge-inherited paradigm, the pre-trained and recycled INN does not have sufficient generalization ability to cope with the large-scale and shape-unset metasurface design. To prove this in detail, we further apply transfer learning for the same inverse design task of the aperiodic metasurface (target task) in Fig. 2a of the manuscript, and the design of Panel A is regarded as the source task. The training results are shown in Fig. S12, where the loss for source task converges well with high accuracy while the loss for target task is non-convergent under a serious underfitting state.


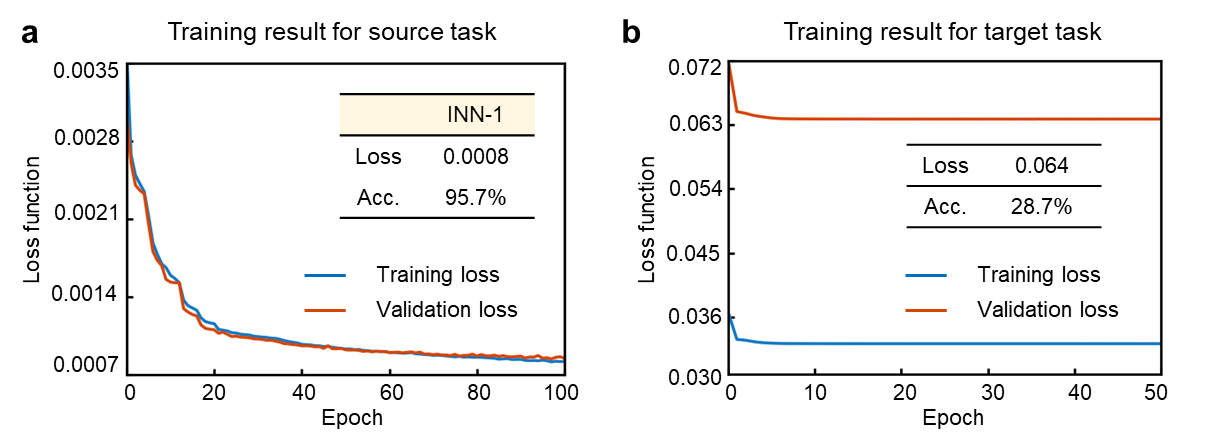


**Figure S12 | Training results of transfer learning.** Training results for (**a**) source task (inverse design task for Panel A) and (**b**) target task (design task of the aperiodic metasurface in Fig. 2a).

**Supplementary Note 6: Evolutive** **antenna theory for tilted metasurfaces**

In antenna theory, each meta-atom element is regarded as an independent radiation source, and the far-field for metasurface is calculated by combining the contributions of all meta-atom elements. Under the normal incidence of plane waves, the far-field function scattered by the metasurface is expressed as follows:

$f\left( \theta, \varphi\right)=\sum_{m=1}^{M} \sum_{n=1}^{N} U_{mn}exp\{ikD[\left( m-1 \right)sin\theta cos\varphi+\left( n-1 \right)sin\theta sin\varphi]\}$ (S3)

where $\theta$ and $\varphi$ are the elevation and azimuth angles of an arbitrary direction, respectively, and $U_{mn}$ is the complex voltage of the unit cell located at $(m, n)$. $M$($N$) is the number of unit cells along the $x$($y$) axis, and $m$($n$) represents the $m$-th($n$-th) unit cell inside. $k$ represents the wavenumber. For tilted “panel” metasurfaces, the inclined array formed by the x-axis as the rotation axis and assuming the panel rotates at an angle of $\alpha$ in the $-\varphi$ direction (taking the x-axis as the rotation axis), as shown in Fig. S13, the modified far-field function is expressed as follows:

$f\left( u, v \right)=\sum_{m=1}^{M} \sum_{n=1}^{N} U_{mn}exp\{ikD[\left( m-1 \right)u+\left( n-1 \right)vcos\alpha-(n-1)\sqrt{1-u^{2}-v^{2}}sin\alpha]\}$ (S4)

Further, if adding the y-axis as another rotation axis and the panel rotates at an angle of $\beta$ in the $-\varphi$ direction, the modified far-field function can be written as:

$f\left( u, v \right)=\sum_{m=1}^{M} \sum_{n=1}^{N} U_{mn}exp\{ikD[\left( m-1 \right)ucos\beta-\left( n-1 \right)vcos\alpha-\sqrt{1-u^{2}-v^{2}}(sin\alpha(n-1)+sin\beta(m-1)]\}$

(S5)

where $u=sin\theta cos\varphi$, $v=sin\theta sin\varphi$.


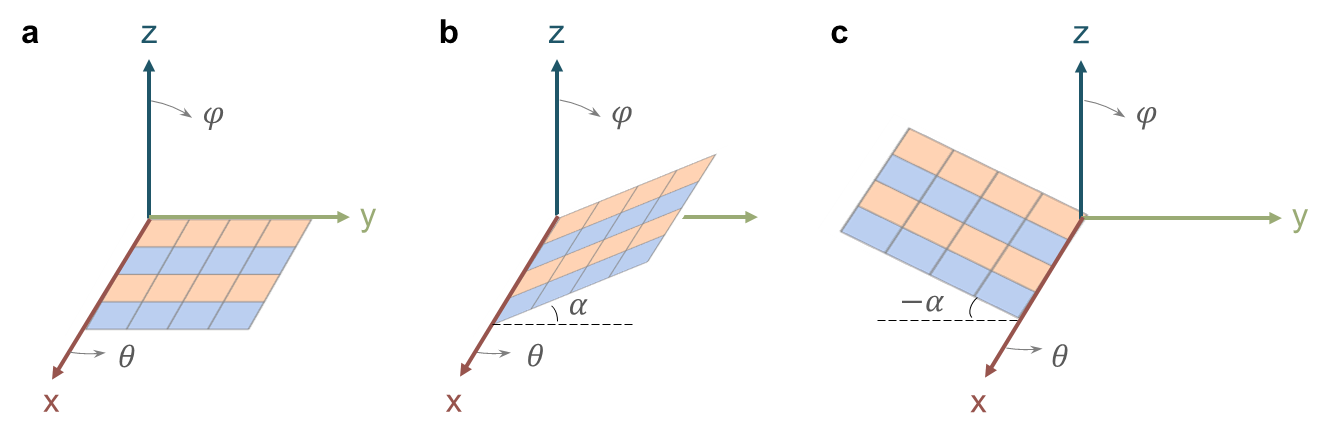


**Figure S13 | Schematic of the inclined meta-surface.** Non-inclined (**a**), forward inclined (**b**) and reverse inclined (**c**) metasurfaces.

**Supplementary Note 7: Another example for free-form metasurface design**

Our method possesses an inheritance-to-assembly mixture scheme that is applicable for a great number of meta-devices and functions. The network can inherit the knowledge from the “parent” metasurface at will, and then disseminate knowledge for the on-demand assembled “offspring” metasurface. It is the high flexibility and free assemblability of this scheme that endows our method with high generality. To further prove this point, we conduct a more complex case to verify the generality, for example, a bird-like shape. To mimic a real bird (Fig. S14a), we construct the bird-like metasurface (Fig. S14b) with 8 “parent” metasurfaces which can be divided into 4 categories (Panel E, Panel G, and the newly-added Panel H, Panel I). Similarly, we train the additional INN-8/9 for Panel H/I and a new SNN for this bird-like “offspring” metasurface. The accuracies for INN-8/9 are 95.4% and 96.7%, respectively, and the training results are shown in Fig. S14c. For this new SNN, the dimension of the input is 4 × 91 × 2, and that of the dual output is 4 × 91 × 8, where the testing accuracy is 96.4% (Fig. S14d). After assembling the corresponding INNs, the terminal tandem accuracy for this newly synthesized neural network reaches 88.6%, further proving the strong generalization ability and universality of our method, even when dealing with complex models and functions.


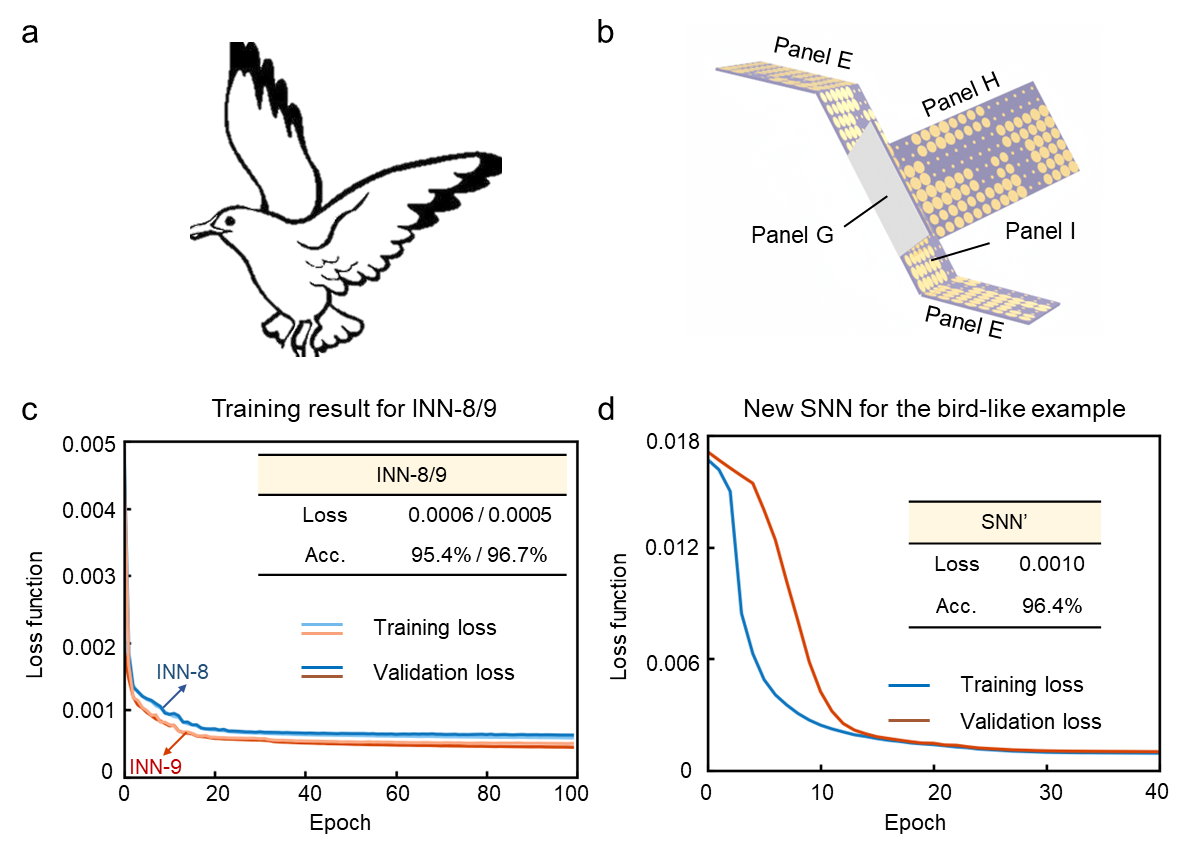


**Figure S14 | A new example of “offspring” metasurface.** To mimic (**a**) a real bird, we construct (**b**) the bird-like metasurface, where the training results for (**c**) INN-8/9 and (**d**) the new SNN prove the generality of our method.

**Supplementary Note 8:** **Experimental measurement and fabrication**

The experiment was carried out in an anechoic chamber, mainly including a transmitting horn antenna and a receiving horn antenna (Fig. S15). The origami metasurfaces of the experiment were made using a curved resin supporter and flexible copper-clad laminate. The curved resin supporter was made using C-UV 9400E, which is an ABS-like SL resin with accurate and durable features (the relative dielectric constant is 3.5) and a thickness of 1.95 mm. The flexible copper-clad laminate was made using Pyralux®AK, which is an all-polyimide composite of polyimide film bonded to copper foil (the relative dielectric constant is 3.4) with a thickness that is 50μm. Each side of the curved resin support and the flexible copper-clad laminate were extended by 4 mm to drill 3 mm small holes, which were further fixed with medium screws.


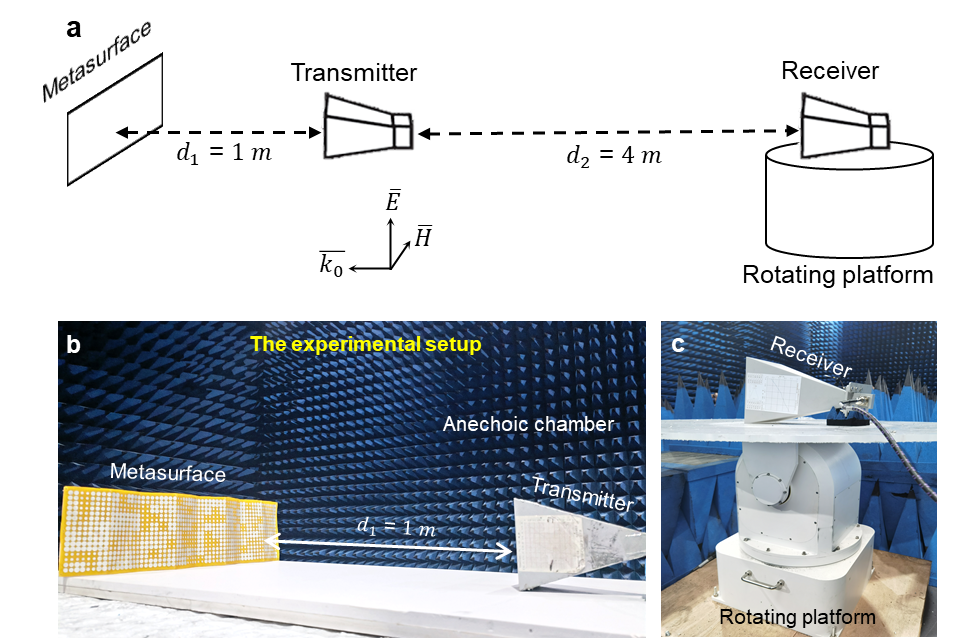


**Figure S15 | Experimental setup of the intelligent origami metasurfaces.** **a**, Schematic diagram of the experimental setup, where (**b**) the transmitting antenna is placed at a distance of 1 m from the metasurface, and (**c**) the receiving antenna is placed on a rotating platform at a distance of 4 m from the metasurface.

**References**

[S1] Al-Saffar, A. A. M., Tao, H. & Talab, M. A. Review of deep convolution neural network in image classification. In *International Conference on Radar, Antenna, Microwave, Electronics, and Telecommunications (ICRAMET)* (2017).

[S2] Ruder, S. An overview of gradient descent optimization algorithms. *arXiv preprint arXiv:1600.04747* (2016).

[S3] Kingma, D & Ba, J. Adam: a method for stochastic optimization. *arXiv preprint arXiv:1412.6980* (2014).
